# Supplementary material for: Chromothripsis during telomere crisis is independent of NHEJ, and consistent with a replicative origin
Source: Genome Res. 2019 May;29(5):737–49. doi: 10.1101/gr.240705.118 (PMC6499312; doi:10.1101/gr.240705.118)
Supplement: Supplemental Material [file supp_gr.240705.118_Supplemental_file_1.zip › contigs/annotated_contigs/DB73/contig.3.DB73_length_365_mean_cov_10.2356164384.docx]

**DB73_length_365_mean_cov_10.2356164384**

```
TAGTTGCTATACTAGTTTACATTACTTTCTTTCTTTTCTTTCTCTTTCTTTCTTTTCTTTCTCTTTCTCTTTCTTTCCTTCCTTCCTTC
 >chr6:131803171-131803352 + E=5e-65 p=2e-02
CTTCCTTCCTTCCTTCCTTCCTTCCTTCTTTCTTTCTTTCTTTCTTTCTTTTCTTTCTTTCTTTCTTCCTTTTCTTCTTTCTTTCTTT|

TCTTTATTTCTTG|ACAGAGTCTTGCTCTGTTGCCCAGGCTGGAGTGCAATGGTGTGATCTCGGCTCACTACAACCTCCACCTTCCAGG
             >chr3:196409514-196409645 + E=2e-52
TTCAAGCGATTCTCCTGCCTCAGCCTCCCGAGTAGCTGGGATTACAGGTGAACACC|GCCACGTCTCACTG|ATTTTTTGTACTTTTAG
                                                                       >chr2:100028516-
TAGAGATGTGGTT100028546 - E
```
